# On the Use of Chloride of Gold in Microscopy

**Published:** 1869-06

**Authors:** Thomas Dwight


On the Use of the Chloride of Gold in Microscopy: *By Thomas Dwight, Jr., M. D.*—Perhaps no re-agent has of late years played so important a part in microscopy as the chloride of gold. By means of it Conheim first demonstrated the terminations of the nerves of the cornea; and since it has been very generally used, particularly in investigations of the nerves. Its application is very difficult, and it is only after a long series of experiments and failures that proficiency is obtained.

Having had considerable experience with this re-agent in the laboratory of Professor Stricker, in Vienna, and having obtained some very satisfactory results, I hope that a few words on its application may not be out of place. The chloride should be dissolved in distilled water, and the solution should never be stronger than the half of one per cent. The object to be examined should be as fresh as possible, and should remain in the fluid from three minutes to perhaps an hour, according to its affinity for the re-agent, during which time it assumes a pale straw color. If the piece be small enough to be readily acted upon, ten or fifteen minutes is almost always sufficient. It is then laid in distilled water, to which just enough acetic acid has been added to give it the faintest possible reaction. In two or three days it will have become purple, verging sometimes on blue, sometimes on red; the latter is the least favorable. The preparation is now enclosed in glycerine, and improves for several days as the color becomes deeper and as the finest fibres are the last to be affected. If the experiment has succeeded, for it sometimes unaccountably fails, the picture presented is one of the most beautiful that can be imagined. The nerves, muscular fibres and fibrous tissue appear black on the purple background. Epithelial cells are also colored, but not so well as by nitrate of silver.

Although the color makes fibres visible which are so fine that they can be seen by no other method, it does not determine their character. To prove beyond all doubt that a minute fibre is a nerve, we must be able to follow it to a larger branch. On a very successful preparation of the cornea of a frog, I observed nerve fibres of such minuteness that with a magnifying power of nearly two thousand diameters it was impossible to follow them to their terminations. I particularly endeavored to verify the connection, asserted by Kühne but not generally accepted, between the nerves and the corneal corpuscles. With every advantage, such a connection is very difficult to prove. I often thought I had found one; but, when examined by a higher power, and placed in different lights, it proved to be only apparent, except in a single instance, and then it was not certain that the fibre in question was a nerve. I mention these facts as proofs of the value of the method, for it is no paradox to say that the better the preparation the more difficult it is to obtain results. As the magnifying power is increased, elements come into view, which, by inferior methods, are never seen; and spaces are discovered between bodies supposed to be in connection. The use of the chloride of gold, however, is not yet thoroughly understood, and offers a large field for original investigation.

